# Effect of the Use of Bovine Appeasing Substance on Immunological, Metabolic, and Oxidative Parameters of Postpartum Dairy Cows: Modulation of the Stress Axis

**DOI:** 10.3390/ani16081185

**Published:** 2026-04-13

**Authors:** Alexandro Fritzen, Guilherme Luiz Deolindo, Luisa Nora, Aleksandro Schafer da Silva

**Affiliations:** 1 Multicenter Postgraduate Program in Biochemistry and Molecular Biology, Santa Catarina State University (UDESC), Lages 88520-000, Brazil; fritzen.vet@gmail.com (A.F.); guilhermeluizd@outlook.com (G.L.D.); luisa.nora22@gmail.com (L.N.); 2 Department of Animal Science, Santa Catarina State University (UDESC), Chapecó 89815-630, Brazil

**Keywords:** stress, immunometabolism, dairy cows, transition period

## Abstract

The responsiveness of the stress axis is fundamental for maintaining health and sustaining productive performance; however, the effect of modulating this stress axis with bovine appeasing substance and its effects on biochemical, immunological, oxidative parameters and uterine involution have not been determined, which are the objectives of this experiment. To elucidate these questions, Holstein cows, from the prepartum to lactation period in a cross-ventilation system, received an application of a bovine appeasing substance (treated group) and a 0.9% saline solution (control group) at the time of calving, and blood samples were collected on calving day and on days 3, 7, 14 and 21 postpartum for analysis. Modulation of the stress axis by bovine appeasing substance increased magnesium levels on days 7 and 14 postpartum, with a reduction in fructosamine levels on days 3, 7, 14, and 21 postpartum. A reduction in ferritin levels, an acute-phase protein, and a reduction in interleukin 1 beta and interleukin 6 were also observed, demonstrating an anti-inflammatory effect in cows of the treated group. Creatine kinase activity decreased on day 21 postpartum in cows treated with bovine appeasing substances. An increase in cholinesterase activity on day 7 and a marked decrease on day 21 postpartum in treated cows were observed compared to the control. This was accompanied by a reduction in beta-hydroxybutyrate levels on day 7 and a reduction in reactive oxygen species levels on day 14 in animals of the treated group, indicating modulation of ketogenesis and reduced oxidation through an anti-inflammatory effect. Mean uterine thickness was also affected by the bovine appeasing substance, with a lower mean thickness on day 21 postpartum in treated cows. Modulation of the stress axis by the bovine appeasing substance reduces inflammation, improving energy dynamics and reducing oxidation, thus facilitating tissue repair associated with postpartum uterine involution in dairy cows.

## Simple Summary

This study investigated whether a natural product called bovine appeasing substance can help dairy cows cope with stress after calving. The period after calving is challenging for cows, as they experience inflammation, metabolic changes, and increased stress, which can negatively affect health, milk production, and reproduction. In this experiment, cows received the substance at calving, and their blood and health indicators were monitored during the first three weeks after birth. Cows treated with the appeasing substance showed lower stress levels, reduced inflammation, and improved metabolic balance compared to untreated cows. They also had lower levels of harmful molecules associated with oxidative stress and showed signs of better recovery of the uterus after calving. In addition, treated cows produced more milk during early lactation. Overall, the results suggest that reducing stress through this substance can improve health, recovery, and productivity in dairy cows after calving. This approach may offer a practical strategy to enhance animal welfare and efficiency in dairy production systems.

## 1. Introduction

The postpartum period is marked by significant challenges associated with milk production, uterine involution, and inflammatory resolution, determining the success of lactation and the maintenance of reproductive performance in dairy cows [1]. This period is marked by increased concentrations of β-hydroxybutyrate (BHB) and non-esterified fatty acids (NEFAs) that interfere with cortisol responsiveness patterns, which show elevated baseline levels in studies with challenges by these metabolites and lipopolysaccharides from bacteria and a reduction in their pulsatile release pattern [2,3]. Cortisol levels are related to the fertility of cows postpartum, with higher levels of this hormone observed in animals with clinical diseases and poor reproductive performance [4]. However, the mechanistic relationship is still speculative, with studies showing that animals with inflammation and reduced liver function have low cortisol levels after application of adrenocorticotropic hormone (ACTH), suggesting reduced adrenal responsiveness [5].

Maintaining health during the transition period (three weeks before and three weeks after calving) depends closely on immune regulation and control of postpartum inflammation [6]. It has been observed that conditions such as ketosis, hypocalcemia, and fatty liver are associated with underlying inflammation [7,8]. Cortisol plays a central role in controlling inflammation and inflammatory resolution [9]. In non-bovine models, exposure to chronic stress has been observed to reduce sensitivity to this hormone, causing low-grade inflammation and predisposing to metabolic disease [10]. Interestingly, in dairy cows, the transition period is marked by a reduction in the expression of cortisol receptors in polymorphonuclear cells, reducing the signaling capacity of the stress axis with the immune system of dairy cows [11], which allows for greater persistence of inflammation; however, this hypothesis requires testing.

The use of bovine appeasing substance (BAS) to reduce the response of stress is accompanied by a decrease in circulating cortisol levels, with an increase in health and fertility [12,13]. BAS is a synthetic analog of the pheromone produced on the teat skin of cattle, which is perceived by the vomeronasal organ of the calf during suckling, leading to an increase in GABA (gamma-aminobutyric acid) levels in the limbic system and a reduction in the stress response [13]. Comprising a mixture of saturated (palmitic acid) and polyunsaturated fatty acids (oleic and linoleic), BAS is perceived by the vomeronasal organ of cattle, reducing amygdala activity and thus reducing the axis stress response [13], with effects on energetic metabolism, although these are not yet fully understood [14]. However, it presents a tool for studying the stress axis in cattle.

The release of cortisol during periods of stress has an anti-inflammatory effect mediated by the interaction with glucocorticoid receptors; however, in states of chronic stress, the anti-inflammatory effect of cortisol is reduced, resulting from a reduction in receptors and the hormone–receptor interaction with chronic inflammation, elevated cortisol levels, and alteration of its pulsatile pattern [5,15]. In postpartum dairy cows, reduced expression of cortisol receptors and altered responsiveness are observed [11], but the effect of BAS on cortisol responsiveness and its effects on inflammation and metabolism in postpartum cows are still poorly investigated. Studies indicate a reduction in stress and cortisol levels when bovine appeasing substances are applied [16], with a decrease in haptoglobin levels, suggesting greater responsiveness [17].

The early postpartum increase in insulin-like growth factor 1 (IGF-1) levels is associated with improved health indicators and reduced aerobic glycolysis in immune system cells, demonstrating a relationship between metabolism and the immune system [18]; however, the effect of cortisol reduction on IGF-1 levels is not yet established. In other species, IGF-1 affects macrophage profiles, acting on the polarization of macrophages towards anti-inflammatory M2 profiles [19].

Maintaining metabolic health and performance is associated with adaptation to lactation, energy metabolism, and postpartum inflammatory resolution [1], supporting the hypothesis that modulation of the stress axis has effects on inflammation and on production, metabolism, and tissue repair in dairy cows. The present experiment aims to evaluate the effect of BAS on metabolism, immune system, oxidative stress, and uterine involution of dairy cows postpartum.

## 2. Materials and Methods

### 2.1. Animals, Facilities and Treatments

For this experiment, 28 Holstein cows housed in a compost barn in a cross-ventilation system with 15 m^2^ of bedding space per cow, an average body weight of 520 kg (standard deviation of 61.55 kg body weight), receiving a total mixed ration (Table 1) in prepartum and postpartum, were randomly divided at calving into two groups of 14 animals with the same proportion of primiparous and multiparous cows in their parity composition. At calving, the cows in the treatment group received 10 mL of BAS (FerAppease^®^, Ouro Fino animal health, Cravinhos, São Paulo, Brazil) divided into 5 mL applied to the neck and 5 mL applied above the snout, as recommended by the manufacturer. After application, the cow and the newborn calf were separated, and blood was collected via the coccygeal vein in a clot activator tube to obtain serum. The animals in the control group followed the same procedures as the treated group, receiving 0.9% saline solution instead of BAS. The animals subjected to the experiment did not present any clinical complications.

Production data were provided by software reports (GEA^®^Model Dairy Plan, version C21) associated with collar data collection (GEA^®^ CowScout), evaluating milk production through the milking and collar system three times a day, from day 3 to day 63 postpartum. Blood samples were collected via coccygeal vein/artery puncture using a needle and vacuum tubes with clot activator to obtain serum and were collected on days 3, 7, 14, and 21 postpartum from animals in the treated and control groups. On day 7 postpartum, BHB levels were evaluated using a handheld meter (Optium Xceed, Abbott Diabetes Care Inc., Alameda, CA, USA and β-ketone strips), with the results determined in mmol/L. After collecting, the serum samples were separated and stored in duplicate in Eppendorf tubes, then frozen at −8 °C for later analysis.

### 2.2. Laboratory Analyses

For serum biochemical analyses, an automated analyzer (Model EXC 200; Zybio^®^ Inc., Chongqing, China) and commercial kits (Analisa^®^, Belo Horizonte, Minas Gerais, Brazil) were used. The variables analyzed were the activities of the enzymes creatine kinase and cholinesterase measured in U/L, as well as the levels of total protein (g/dL), albumin shown in g/dL, cholesterol, calcium, magnesium, urea and ferritin presented in mg/dL, and fructosamine measured in µmmol/L. Globulin levels were obtained mathematically (total protein–albumin) in mg/dL. The protocol followed the recommendations of the manufacturer of the commercial kits.

Serum levels of IL-1β and IL-6 cytokines were measured using commercial kits (USCN Life Science Inc., Wuhan, China) and an enzyme-linked immunosorbent assay (Chem Well, Awareness Technology Inc., Palm City, FL, USA) to determine their concentrations in pg/mL. Serum cortisol (Immulite Cortisol, Siemens Healthcare Diagnostics, Tarrytown, NY, USA) and insulin-like growth factor I-IGF-I (Immulite IGF-1, Siemens Healthcare Diagnostics, Tarrytown, NY, USA) were measured with immunoassay tests that implemented the chemiluminescence method using a commercial kit, with the methodology also being described by Baldacim et al. [20]. Non-esterified fatty acids (NEFA) were analyzed using Randox^®^ kits (FA115—Colorimetric Method and RB1007—Enzymatic Method, Antrim, UK) [21].

ROS analysis in serum was performed using a fluorimetric protocol established by Lebel et al. [22], where 10 µL of serum was incubated with the same amount of 2′,7′-dichlorofluorescein diacetate (DCFH-DA, 7 µM) and 240 µL of sulfate-buffered saline (PBS). Incubation was carried out for 30 min at 37 °C, followed by evaluation of the oxidation product of DCFH-DA, dichlorofluorescein (DCF). Fluorescence emission intensity was measured at 525 nm emission and 488 nm excitation (Varioskan™ LUX, Thermo Fisher Scientific, Waltham, MA, USA).

For TBARS analysis in serum, a lipid peroxidation indicator was used, following the method described by Jentzsch et al. [23]. To evaluate TBARS, the reaction of thiobarbituric acid with serum samples was used, which in the presence of malondialdehyde results in a pink product that can be read at 532 nm. Twenty µL of the sample was homogenized with 55 µL of distilled water, 100 µL of orthophosphoric acid (0.2 M) and 25 µL of thiobarbituric acid (0.1 M). After 45 min of incubation at 37 °C, spectrophotometric readings were performed. Adenosine deaminase activity in serum was evaluated using a spectrophotometric method as described by Giusti and Gakis [24], with the result expressed in U/L.

### 2.3. Uterine Thickness Assessment

On day 21 postpartum, the cows were restrained and immobilized in a cubicle, where rectal palpation was performed with the aid of gloves and gel. After locating the uterus and determining the position of the uterine horns, an ultrasound image was performed using an ultrasound (DP-10 Power, Mindray Medical International Ltd., Shenzhen, China) and an endorectal probe, 5 cm cranial to the intercornual ligament, for imaging and evaluating the thickness (serosa to serosa) of the uterine horn. The same procedure was performed for the contralateral uterine horn, and the arithmetic mean of the uterine thickness was calculated and tabulated for subsequent statistical analysis.

### 2.4. Statistical Analysis

All data were analyzed using the ‘MIXED’ procedure of SAS (SAS Inst. Inc., Cary, NC, USA; version 9.4), using Satterthwaite approximation for denominator degrees of freedom in fixed effects testing. Serum biochemistry, protein profile, cytokines, oxidative stress and enzymes data were analyzed as repeated measures and tested for fixed effects of treatment, day, and treatment × day interaction, using the animal (treatment) as a random effect. Data of BHB and uterine thickness were analyzed for fixed effects of treatment and the animal (treatment) as a random effect. Means were compared using the PDIFF method, and all results were presented as least squares means (LSMEANS) followed by the standard error of the mean (SEM). Data were considered statistically significant if *p* ≤ 0.05.

## 3. Results

**Figure 1:**
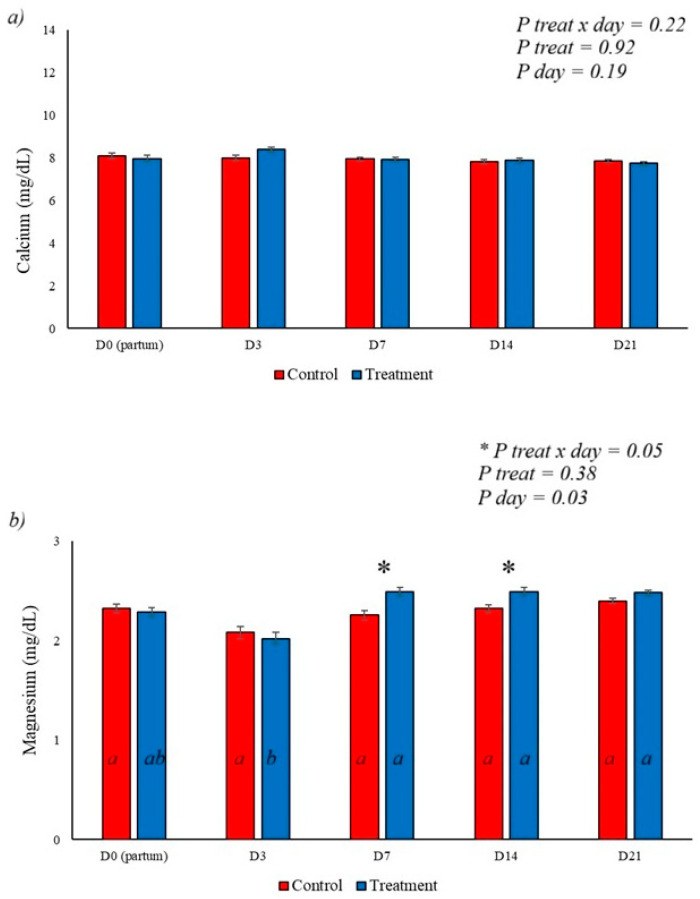
(**a**) shows serum calcium levels during the experimental period, demonstrating no effect of treatment, day, or interaction between treatment and day; (**b**) demonstrates the effect of BAS on serum magnesium levels. The use of BAS increased magnesium levels on days 7 and 14 of the experiment, with the lowest serum level observed on day 3 postpartum. Different letters represent statistical differences in the day interaction. * Represents the interaction between treatment x day. The different letters (a, b) illustrate the effects of day, showing the difference over time within each group.

This experiment demonstrates the positive effect of BAS on metabolic, immunological, oxidative, productive, and uterine markers in postpartum dairy cows. The effect of BAS on serum calcium levels was not observed, with no significant variation between days or groups, as shown in Figure 1a. Levels of serum magnesium were lower on day 3 postpartum in the group that received BAS; however, the opposite effect was observed on days 7 and 14 postpartum (*p* = 0.05), where the treated animals showed higher magnesium levels than the animals in the control group, with this difference disappearing on day 21 postpartum (Figure 1b).

**Figure 2:**
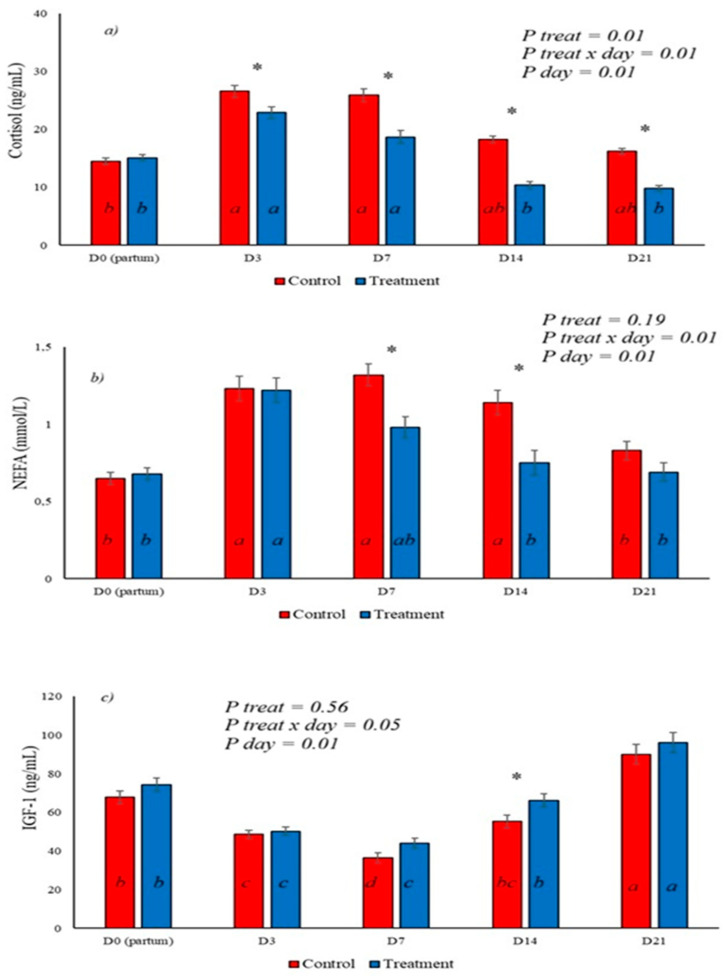
Effect of BAS treatment on serum cortisol, NEFA, and IGF-1 levels in postpartum cows. (**a**) shows a reduction in cortisol levels in the BAS-treated group on days 3, 7, 14, and 21. (**b**) shows the effect on serum NEFA levels, with a reduction on days 7 and 14 in treated animals. (**c**) shows the effect of BAS on IGF-1, which increased early in the postpartum period in treated animals, with higher levels on day 14 compared to the control. * Represents the interaction between treatment x day. The different letters (a, b, c, d) illustrate the effects of day, showing the difference over time within each group.

Serum cortisol levels showed an increase from calving to day 3, with a progressive decrease on days 7, 14, and 21. Levels were lower in the group treated with BAS from day 3 postpartum onwards, and levels remained lower on days 7, 14, and 21 (Figure 2a), demonstrating a marked effect of BAS on cortisol levels in postpartum dairy cows. NEFA levels increased after calving, with higher serum levels on days 3, 7, and 14, returning to lower levels on day 21, as shown in Figure 2b. In animals that received BAS, a reduction in NEFA levels was observed on days 7 and 14 postpartum, demonstrating a reduction in the mobilization of reserves earlier than in the control group (Figure 2b). IGF-1 showed the lowest serum concentrations on days 3 and 7 postpartum (Figure 2c), with a progressive increase in its levels on days 14 and 21, being highest on day 14 in the group that received BAS, which demonstrates an earlier increase in its serum levels in the treated animals.

**Figure 3:**
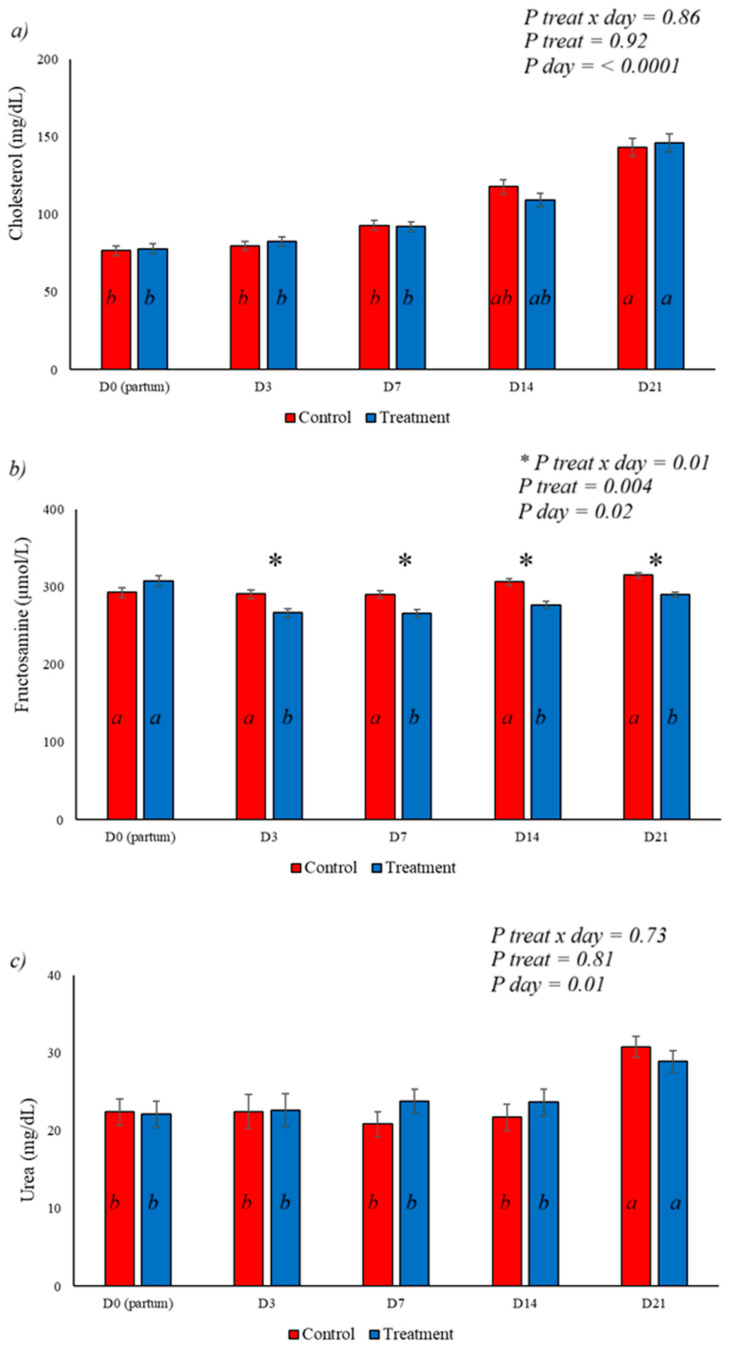
(**a**) demonstrates the progressive increase in cholesterol levels from the day of delivery to the 21st day, but without interaction between day or treatment; (**b**) shows the effect of BAS on fructosamine levels, which were lower in the treated group on days 3, 7, 14, and 21 postpartum, with day and treatment x day interactions; (**c**) shows serum urea levels, with no interaction between treatment and treatment x day, but with an increase in levels on day 21 when compared to the others. Different letters represent statistical differences in the day interaction. * Represents the interaction between treatment x day. The different letters (a, b) illustrate the effects of day, showing the difference over time within each group.

In the postpartum period, an increase in cholesterol levels was observed, with the highest levels on days 14 and 21 of the experiment (Figure 3a); however, this behavior was not influenced by the BAS treatment. This same behavior was observed at urea levels, with a marked increase in serum urea on day 21 postpartum, but without any effect from the treatment (Figure 3c). The BAS treatment showed a marked effect on fructosamine levels, with a reduction in serum levels in treated animals in the days following parturition (Figure 3b). A day-by-day and treatment-by-day interaction was observed for fructosamine.

**Figure 4:**
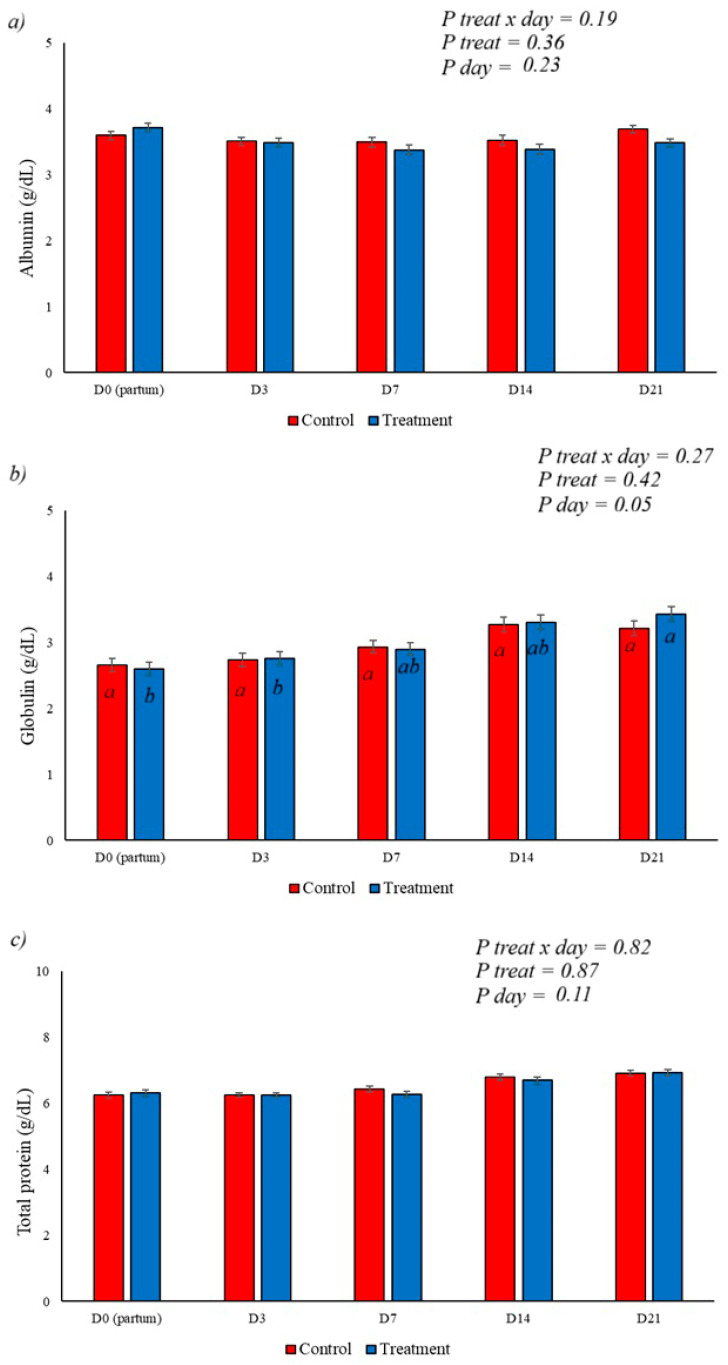
(**a**–**c**) demonstrate the behavior of serum levels of albumin, globulins, and total proteins, respectively. No interaction of treatment, day, or treatment x day was observed for the variables albumin and total proteins, with a day effect for globulins, which showed an increase after parturition. The different letters (a, b) illustrate the effects of day, showing the difference over time within each group.

When we analyzed the proteinogram, there was no statistically significant difference in albumin and total protein levels. A day effect was observed for globulin levels, but there was no effect from the BAS treatment, as shown in Figure 4a–c.

**Figure 5:**
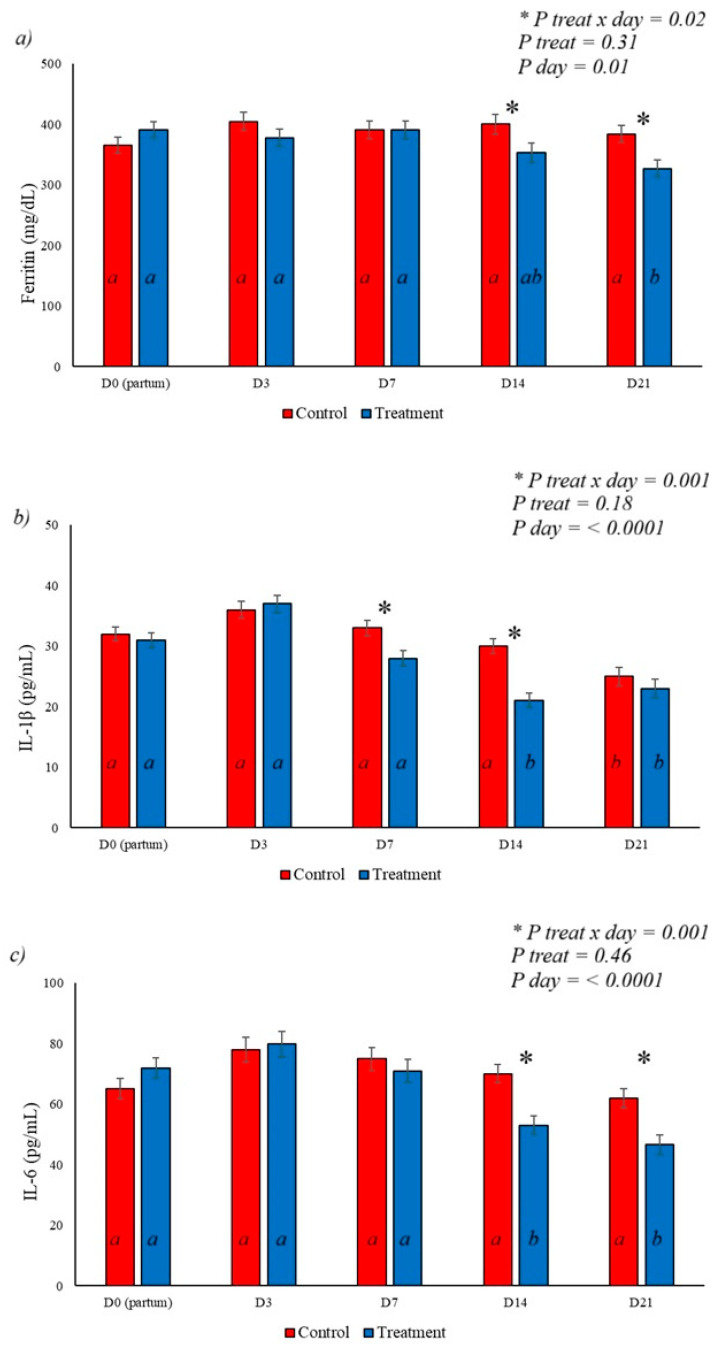
Effect of BAS application on immunological parameters of postpartum dairy cows. (**a**) shows the effect of the treatment on serum ferritin levels, with a reduction on days 14 and 21 postpartum in the treated group; (**b**) demonstrates the reduction in IL-1B levels in the treated group, with a marked effect on days 7 and 14 postpartum. The IL-6 levels shown in (**c**) demonstrate a reduction in the treated group on days 14 and 21 postpartum, demonstrating the effect of BAS on this variable. Different letters represent statistical differences in the day interaction. * Represents the interaction between treatment x day. The different letters (a, b) illustrate the effects of day, showing the difference over time within each group.

When analyzing immunological parameters, it was observed that the application of BAS in the treated group promoted a reduction in ferritin levels on days 14 and 21 postpartum, with a treatment x day interaction and a day effect, as shown in Figure 5a. IL-1β levels showed a marked reduction on days 7, 14, and 21 of the experiment (Figure 5b), with a day effect on this variable (*p* < 0.001) with lower levels of this cytokine in the treated group on days 7 and 14, demonstrating a treatment x day effect (*p* = 0.001). Serum IL-6 levels showed a treatment x day effect and a day effect on this variable, with a decrease in levels recorded in the treated group on days 14 and 21 postpartum, as shown in Figure 5c.

**Figure 6:**
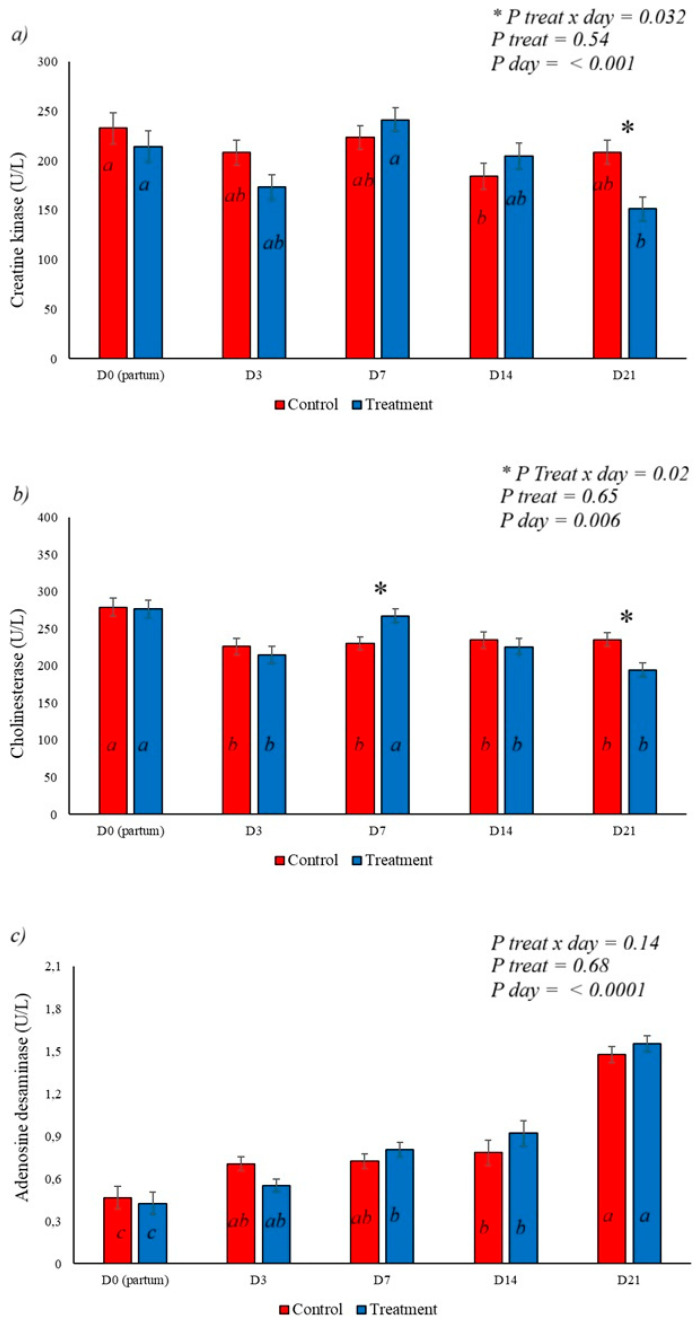
Effect of BAS treatment on serum enzymes. (**a**) demonstrates a reduction in creatine kinase activity on day 21 postpartum in the treated group, while (**b**) shows the effect of the treatment on cholinesterase activity, with an increase in activity on day 7 and a reduction on day 21 postpartum in the treated group. (**c**) shows the behavior of adenosine deaminase, which showed a progressive increase in its serum activity after delivery, but without an effect from the treatment. Different letters represent statistical differences in the day interaction. * Represents the interaction between treatment x day. The different letters (a, b, c) illustrate the effects of day, showing the difference over time within each group.

Creatine kinase (CK) levels were elevated at the time of delivery, decreasing on subsequent days, demonstrating a treatment x day and day effect, with the lowest levels of this enzyme on day 21 in the group that received BAS at delivery (Figure 6a). Cholinesterase showed distinct behaviors (Figure 6b), with increases in its activity on day 7 postpartum and a reduction on day 21 in the treated group, with a day (*p* = 0.006) and treatment x day effect observed for this variable (*p* = 0.02). Adenosine deaminase (ADA) showed a progressive increase in activity after delivery (*p* < 0.0001), with no effect of BAS use on its activity, as demonstrated in Figure 6c.

**Figure 7:**
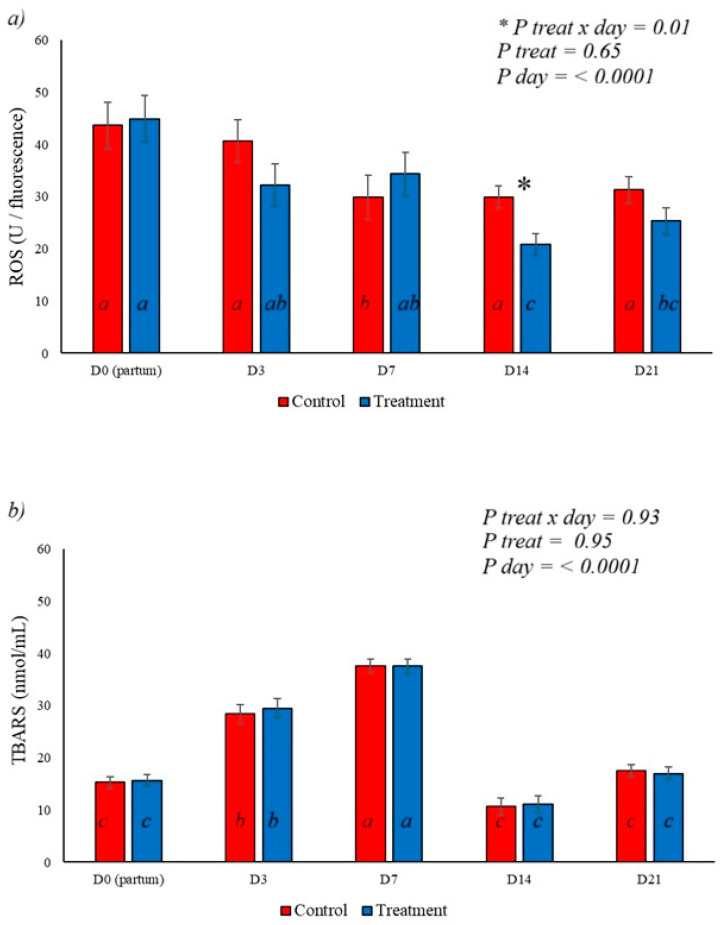
Effect of BAS on oxidative parameters. (**a**) demonstrates the effect of the treatment on reactive oxygen species (ROS), with the treatment x day effect showing a reduction in ROS on day 14 when compared to the control. A day effect was also observed on this variable, with a progressive reduction in its levels after parturition. (**b**) shows the behavior of thiobarbituric acid reactive substances (TBARS), with a progressive increase in their levels until the 7th day postpartum and a consequent reduction to levels like those at parturition on days 14 and 21, with no treatment or treatment x day effect for this variable. Different letters represent statistical differences in the day interaction. * Represents the interaction between treatment x day.

ROS levels were affected by BAS application, with a reduction on day 14 postpartum in the treated group (*p* = 0.01), demonstrating a treatment x day interaction. A day effect was observed, with a decrease in ROS after calving, as shown in Figure 7a. TBARs were not affected by BAS application, with an increase in levels observed, peaking on day 7 postpartum and returning to levels observed at calving on days 14 and 21 of the experiment (Figure 7b), demonstrating a day effect on this variable (*p* <0.0001).

**Figure 8:**
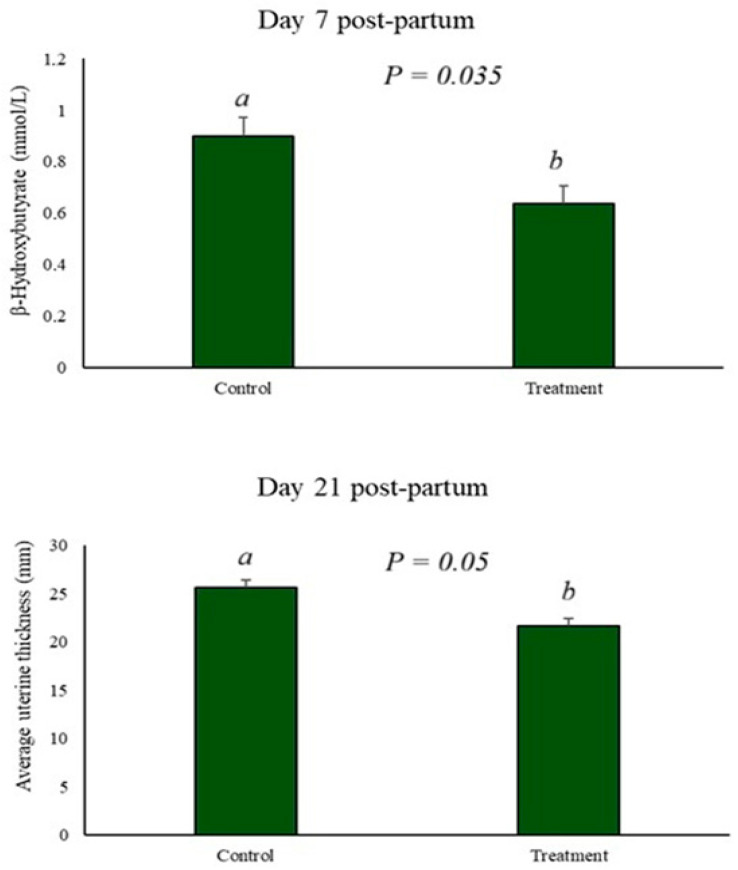
Effect of BAS application on BHB levels and mean uterine thickness, demonstrating a reduction in BHB levels at 7 days postpartum and lower uterine thickness at 21 days postpartum in treated animals. Letters represent differences between groups.

The evaluation of ketone bodies performed on day 7 postpartum showed an effect of BAS treatment, with lower serum BHB levels in the treated group (*p* = 0.03). On day 21 postpartum, evaluation of average uterine thickness (AUT) demonstrated an effect of BAS treatment on the mean AUT in the treated animals (*p* = 0.05). Figure 8 illustrate the BHB and AUT levels, respectively.

**Figure 9:**
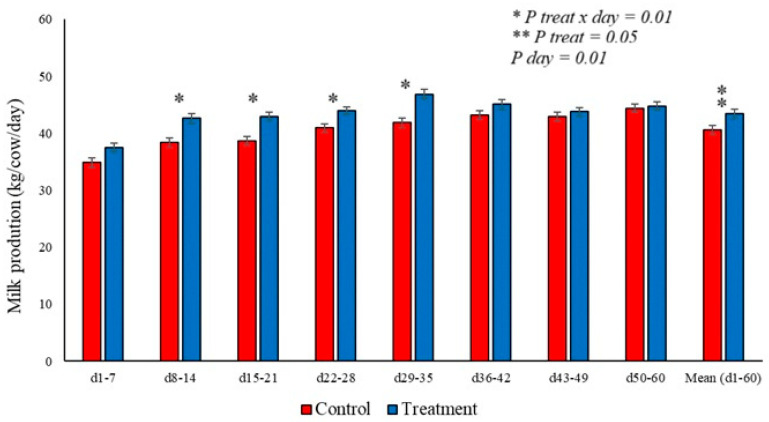
Effect of BAS treatment on milk production in dairy cows at calving, divided into means represented by columns. Red columns represent the control group, and blue columns represent the treated group. Animals in the treated group showed higher production from days 8–14 to days 29–35, with a higher average for the period than the control group. * Represents a statistically significant difference (*p* < 0.05). ** Represents a statistically significant difference (*p* < 0.01).

Milk production was positively affected in the treated group, with higher cumulative production during the periods from days 8 to 14 and 29 to 35, which was reflected in higher averages in the animals treated, as shown in Figure 9. The animals in the treated group produced, on average, 2.7 L per day more milk cumulatively during the evaluation period (d1-60) than the control group, as shown in Figure 9, across columns 1 to 60 of the graph.

## 4. Discussion

**Figure 10:**
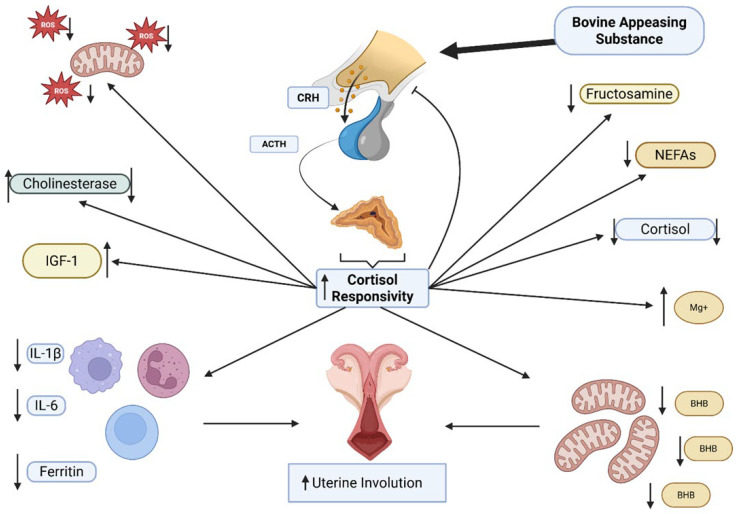
Effect of modulation of the stress axis with BAS. Modulation reduced levels of fructosamine, BHB, ROS cortisol, NEFAs, and inflammatory cytokines such as IL-1β and IL-6, with an increase in cholinesterase activity followed by a reduction. IGF-1, milk production and magnesium levels increased, and a reduction in average uterine thickness was also observed, indicating improvements in uterine involution.

Maintaining mineral metabolism homeostasis during the transition is influenced by factors such as milk production, diet, and immune status, and is central to determining health and the genesis of diseases with hypocalcemia and hypomagnesemia [7,25]. This experiment demonstrated no effect of BAS on serum calcium levels, which remained within normocalcemic ranges. Observed calcium levels were associated with the use of anionic diets during the 21 days preceding parturition. Anionic diets, combined with adequate vitamin D3 supplementation and inflammation control, are associated with the prevention of postpartum hypocalcemia [25,26]. In cattle, the maintenance of magnesium homeostasis is achieved through absorption in the rumen and renal excretion [27]. The relationship between increased magnesium levels and protection against stress has been verified, with increased cortisol levels responsible for the increased renal excretion of this mineral due to increased aldosterone levels [28]. The present experiment demonstrated an increase in serum magnesium levels on days 7 and 14 postpartum in animals treated with BAS (Figure 1b), supporting a reduction in stress responsiveness demonstrated by a reduction in serum cortisol levels (Figure 2a and Figure 10). A reduction in serum cortisol levels is also observed in beef cattle that received BAS [16].

After calving, milk production is accompanied by an increase in lipolysis mediated by hormones such as GH, cortisol, and catecholamines, activating hormone-sensitive lipases (HSLs) in adipose tissue and raising serum NEFA levels [29]. The reduction in lipolysis is promoted by insulin, which leads to a reduction in HSL activity and a reduction in NEFA levels, while increasing IGF-1 [30]. However, in the postpartum period, peripheral insulin resistance and a reduction in insulin receptors in hepatocytes uncouple the somatotropic axis, reducing IGF-1 levels [31,32]. In cows treated with BAS, cortisol levels showed a significant reduction from day 3 to day 21 of the experiment, accompanied by an earlier reduction in NEFA levels on days 7 and 14 and higher serum IGF-1 levels on day 14, indicating a reduction in lipolysis and a recoupling of the somatotropic axis due to a reduction in the effect of cortisol on insulin responsiveness. Studies demonstrate that exposure of tissues to glucocorticoids reduces the expression of insulin receptors and impairs the action of this hormone [33]. This reinforces the possibility of improved insulin responsiveness, as treated animals showed a marked reduction in cortisol and an anticipated increase in serum IGF-1 levels, but this is not tested in this experiment.

Cholesterol plays an important role in lipid transport, representing a supply of fatty acids to the mammary gland in the form of very low-density lipoproteins (VLDL) and high-density lipoproteins (HDL), and is involved as a substrate for steroidogenesis and regulation of membrane fluidity [34]. This experiment demonstrated a marked increase in serum cholesterol levels on days 14 and 21 postpartum compared to calving, associated with the mobilization of reserves and transport of lipids [34]. Lipolysis is controlled by hormones such as insulin, with peripheral resistance to this hormone associated with inflammation, resulting in metabolic disorders and an inability to maintain normoglycemia [35]. The use of BAS reduced BHB levels on day 7 postpartum (Figure 8 and Figure 10), demonstrating improved energy homeostasis, with a consequent marked reduction in fructosamine in the treated group, demonstrated in Figure 3b. Fructosamine levels reflect glycemia, indicating the ability to maintain normoglycemia and preserved insulin regulation [36,37]; however, insulin responsiveness levels were not tested and are subject to speculation.

The peripartum period is marked by a reduction in serum globulin concentration, associated with the transfer of gamma globulins to colostrum, which then increases in the days following parturition [38]. Albumin levels decrease during this period in cases of liver impairment or diseases that cause serum protein loss [38,39]. The use of BAS did not affect serum protein behavior and had no effect on urea levels, which in this experiment showed the highest levels on day 21 postpartum, possibly reflecting protein catabolism and the conversion of amino groups into urea via the ornithine cycle or increases in dry matter intake [40,41].

In the postpartum period, uterine involution, associated with metabolic stress, constitutes potent inflammatory stimuli, with an increase in pro-inflammatory cytokines and positive acute-phase proteins that disrupt dry matter intake [42] and increase lipolysis [43]. In this context, maintaining cortisol responsiveness plays a central role in controlling inflammatory tone and initiating inflammatory resolution [9]. Elevated basal cortisol levels associated with chronic stress are responsible for reducing the expression of this hormone’s receptors in immune system cells [11,15]. The use of BAS reduced serum levels of IL-1β and IL-6, with an early reduction in these cytokines postpartum compared to the control group, demonstrating a positive effect on inflammation resolution.

The increase in cytokines such as IL-1β, IL-6, and TNF-α is associated with increased synthesis of positive acute-phase proteins in cattle [44], with ferritin, a protein related to iron homeostasis and induced by inflammation, acting as an inflammatory marker [45]. Studies have demonstrated a correlation between serum ferritin levels and inflammatory states such as mastitis or stress [45,46], with the application of BAS reducing serum levels of this acute-phase protein on days 14 and 21 in the treated group, accompanied by a reduction in IL-6 and IL-1β also observed in this experiment (Figure 5a–c), indicating anti-inflammatory activity (Figure 10) and accompanied by a reduction in serum cortisol levels, allowing speculation about increased sensitivity to cortisol.

Creatine kinase (CK), an enzyme responsible for creatine phosphorylation and part of the cellular energy homeostasis system [47], showed higher levels at the time of calving with a progressive reduction until the 21st day postpartum. The effect of BAS on serum CK was observed and was possibly associated with a reduction in inflammation and leakage of this enzyme.

Cholinesterase activity is crucial for regulating acetylcholine levels [48], controlling its availability and consequently its binding to its alpha 7 nicotinic acetylcholine receptor (α7 nAChR) and promoting an anti-inflammatory response [49]. BAS application demonstrated an effect on cholinesterase, which curiously showed higher levels on day 7 in treated animals and lower levels in the same group on day 21 postpartum (Figure 6b and Figure 10). The increased cholinesterase activity on day 7 allows for an inflammatory response and the elimination of bacterial uterine contents from parturition; however, after this phase, the reduction in inflammation allows for tissue repair and inflammatory resolution, key factors in uterine involution [50], but this is a speculative pathway and must validated in new works.

Adenosine deaminase, an enzyme of the purinergic system, is associated with the degradation of adenosine, which has anti-inflammatory effects and is involved in orchestrating the resolution of inflammation [9]. In cattle, an increase in its activity has been observed in hypocalcemia states [51], and this experiment demonstrated a progressive increase in its activity after calving, although treatment with BAS showed no effect on its activity.

Increased inflammation is associated with an elevated production of free radicals, which catalyze the activation of NLRP3 and the release of cytokines [52,53], resulting in tissue damage. It was observed that the application of BAS reduces serum ROS levels (Figure 7a), associated with a reduction in inflammatory cytokines and average uterine thickness on day 21 postpartum (Figure 8). Lower levels of ROS and inflammation allow for tissue repair and uterine involution, maintaining reproductive health postpartum (Figure 10).

## 5. Conclusions

This experiment demonstrates the positive effect of BAS on metabolic, immunological, oxidative, and uterine health parameters in postpartum cows. Cows treated with BAS showed higher magnesium levels, accompanied by a reduction in cortisol, NEFAs, fructosamine, inflammatory cytokines, and ferritin, indicating a marked anti-inflammatory effect associated with improved responsiveness of the stress axis and sensitivity of cortisol. Cholinesterase increased on the seventh day and decreased on the twenty-first day postpartum, accompanied by a reduction in creatine kinase, which points to inflammatory resolution that may be reinforced by the lower average uterine thickness and first increase in IGF-1 levels of the treated cows. The modulation of the stress axis by BAS reduced ROS and ketogenesis, resulting from improved metabolic efficiency associated with cortisol regulation.

## Figures and Tables

**Table 1:** Chemistry composition of prepartum and postpartum diets.

Parameter	Prepartum Diet *	Postpartum Diet
Neutral detergent fiber (NDF; % DM)	39.3	31.6
Acid detergent fiber (ADF; % DM)	25.0	19.2
Metabolizable energy (ME; Mcal/Kg)	2.44	2.41
Crude protein (CP; % DM)	13.5	18.5
Non-fibrous carbohydrates (NFC; % DM)	38.8	41.8
Starch (% DM)	23.7	26.3
Ethereal extract (EE; % DM)	2.4	4.00
Calcium (Ca; %DM)	1.14	0.84
Phosphorus (P; % DM)	0.30	0.37
Magnesium (Mg; % DM)	0.24	0.31
Potassium (K; % DM)	1.06	1.13
Vitamin A (kIU/Kg)	16.1	4.5
Vitamin D3 (kIU/Kg)	6.7	1.3
Vitamin E (IU/Kg)	40.1	35.7
Dietary cation–anion difference (DCAD; meq/100 g)	−11.5	+25.5

* The prepartum diet was provided for the animals during the last 21 days leading to the expected labor date.

## Data Availability

The original contributions presented in this study are included in the article. Further inquiries can be directed to the corresponding author.
